# Integrative Multi-Omics in Biomedical Research

**DOI:** 10.3390/biom11101527

**Published:** 2021-10-16

**Authors:** Michelle M. Hill, Christopher Gerner

**Affiliations:** 1 QIMR Berghofer Medical Research Institute, Herston, Brisbane, QLD 4006, Australia; 2 UQ Centre for Clinical Research, Faculty of Medicine, The University of Queensland, Herston, Brisbane, QLD 4006, Australia; 3 Department of Analytical Chemistry, University of Vienna, 1090 Vienna, Austria; christopher.gerner@univie.ac.at

Genome technologies have revolutionized biomedicine, but the complexity of biological systems cannot be explained by genomics alone. Advances in sequencing and mass spectrometry technologies coupled with methodological and computational innovations are essential in driving multidimensional omics applications.

This Special Issue covers the latest methods and novel findings from integrative analysis of multiple omics datasets to address diverse questions in biology and pathology.

The scene is set with a review article by Lancaster et al. [1], which introduces six players (genome, epigenome, transcriptome, metagenome, proteome and metabolome) that use two different technologies (sequencing and mass spectrometry). After characterizing individual omics data and analytical approaches, considerations for multi-omic study design and data integration methods are discussed.

The contributed research papers span a broad range of studies from clinical cohorts and mouse models to cell-based investigations, thus illustrating the diverse applications of multi-omics.

Two papers applied multi-omics to investigate physiological interventions.

Odenkirk et al. [2] compared the blood lipidome and metabolome in two cohorts of patients undergoing exercise and planned myocardial infarction, respectively, to gain insight on the metabolic pathways underlying the disease and its prevention.

Molendijk et al. [3] applied lipidomic and metagenomic profiling in a dietary model of gastro-esophageal reflux disease and associated esophageal pathology in mice, revealing increased microbiome diversity and a lipidomics signature associated with esophageal inflammation and metaplasia.

Five papers applied multi-omics to diverse cell models, with a study by Niederstaetter et al. [4] highlighting the variability and influence of fetal calf serum (used in culture media)-contained eicosanoids on cellular function, evaluated via proteomics and lipidomics. Neuditschko et al. [5] investigated endometrial pain mechanisms by applying proteomics, metabolomics and eicosanoid profiling to cells derived from endometriotic lesions.

Gillen et al. [6] applied metabolic measurements with secretome profiling to assess the impact of endotoxin (LPS) on macrophages, while Novikova et al. [7] combined transcriptome and proteomic profiling to investigate granulocyte differentiation and discovered HIC1, CEBPB, LYN and PARP1 as potential therapeutic targets in acute myeloid leukemia.

Finally, the paper by Kim et al. [8] illustrates the standardized application of combining drug affinity responsive target stability (DARTS) and mass spectrometry imaging (MSI) to facilitate target protein identification for other existing natural therapeutic compounds.

To wrap up this Special Issue, the comprehensive review article by Howard and Cristea [9] highlights the role of integrative multi-omics in deciphering system-level mechanisms of DNA sensing during viral infections. Following viral infection, protein–protein interactome and protein post-translational modifications drive the remodeling of the cellular transcriptome, proteome and secretome; hence, multi-omic investigations should also include interactome and modification analyses such as phosphoproteome.

In conclusion, multi-omic investigation has become a central technique for deciphering complex biological systems. Continued innovations in technologies, methodologies and applications will enable and support further expansion and integration of multi-omics in future biomedical research.
